# Enriched bovine IgG fraction prevents infections with Enterohaemorrhagic *Escherichia coli* O157:H7, *Salmonella enterica* serovar Enteritidis, and *Mycobacterium avium*

**DOI:** 10.1002/fsn3.1134

**Published:** 2019-07-17

**Authors:** Keiji Funatogawa, Tatsuya Tada, Kyoko Kuwahara‐Arai, Teruo Kirikae, Masao Takahashi

**Affiliations:** ^1^ Tochigi Prefectural Institute of Public Health and Environmental Science Utsunomiya Japan; ^2^ Department of Microbiology Juntendo University School of Medicine Tokyo Japan; ^3^ Aotearoa Co. Tokyo Japan

**Keywords:** bovine IgG‐enriched whey fraction, Enterohaemorrhagic *Escherichia coli* O157:H7, *Mycobacterium avium*, *Salmonella enterica* serovar Enteritidis

## Abstract

A bovine IgG‐enriched whey fraction contains antibodies against various bacterial antigens. We investigated the protective effects of a bovine whey fraction preparation against infections with Enterohaemorrhagic *Escherichia coli* O157:H7, *Salmonella enterica* serovar Enteritidis, and *Mycobacterium avium* in mouse models. After infection with these pathogens, the IgG‐enriched fraction or skim milk was given ad libitum at a 5% solution instead of water. The mice given the IgG‐enriched fraction were significantly resistant to orally challenged EHEC O157:H7 (LD_50_: 4.0 × 10^5^ CFU/mouse) infections compared with the mice given skim milk (LD_50_: <1.5 × 10^2^ CFU/mouse). The mice given the IgG‐enriched fraction were also significantly resistant to orally challenged *S.* Enteritidis (LD_50_: 5.0 × 10^6^ CFU/mouse) infections compared with the mice given skim milk (LD_50_: <2.5 × 10^1^ CFU/mouse). When the mice were nasally infected with *M. avium*, the numbers of the bacteria in lungs of mice given the IgG‐enriched fraction were significantly lower than those given skim milk 2 and 3 weeks after infection. These results strongly indicate that oral administration of the bovine IgG‐enriched whey fraction protects mice against food‐borne infection and also that it partially protects mice against respiratory tract infection.

## 1 INTRODUCTION

Food‐borne infections caused by Enterohaemorrhagic *Escherichia coli* (EHEC) and *Salmonella* spp. are globally important, because of considerable mortality especially in children (WHO: http://www.who.int/news-room/fact-sheets/detail/e-coli and http://www.who.int/news-room/fact-sheets/detail/salmonella-(non-typhoidal). EHEC O157:H7 pathogens cause a server illness with diarrhea, hemorrhagic colitis, and hemolytic uremic syndrome (HUS) (Mead & Griffin, 1998; Siegler, 1995). Vero/Shiga toxin produced by EHEC plays an important role in the progression of hemorrhagic colitis to serious systemic complications such as HUS and neurological manifestations that can cause death (Akashi et al., 1994; Fujii et al., 1994; Richardson et al., 1992; Siegler, 1995). Several outbreaks caused by *Salmonella* spp. have been identified and are most commonly associated with agricultural products (Hanning, Nutt, & Ricke, 2009). Nontyphoidal *Salmonella* infection is usually characterized by a self‐limited gastroenteritis in immunocompetent hosts in developed countries. Typhoid (enteric) fever and its potential complications have a significant impact on children in developing countries.

Several milk preparation products have been reported effective as a prophylactic and treatment for food‐borne infections (Ulfman, Leusen, Savelkoul, Warner, & Neerven, 2018). Bovine colostrum preparations obtained from cows immunized with antigens of various human gastrointestinal infections were called “hyperimmunized milk” (Golay, Ferrara, Felber, & Schneider, 1990). This milk is characterized by high antibody activities against the specific organisms. Several clinical trials indicate that immune cow colostrum can help in shortening the duration of gastrointestinal infections (Ulfman et al., 2018). The second generation is colostrum derived from healthy nonimmunized pasture‐fed cows. Early pioneering studies showed colostrum immunity against *Salmonella* infection in calves (Griffiths, 1969; Royal, Robinson, & Duganzich, 1968), and more recent investigations showed that an immunoglobulin preparation from nonimmunized cows contains high levels of antibodies and neutralizing activity against Vero/Shiga toxin of EHEC O157:H7 (Lissner, Schmidit, & Karch, 1996).

## 2 MATERIALS AND METHODS

### 2.1 Mice

Male Balb/c mice at 8 weeks of age were purchased from Oriental Yeast Co., and were used in the experiments.

### 2.2 IgG‐enriched whey fractions and reagents

The IgG‐enriched whey fraction (IgG25^+^; Aotearoa Co.) were prepared from milk of pasture fed, nonimmunized healthy New Zealand cows by New Zealand Dairy Group by centrifugation to remove fat and condensation of immunoglobulin. The cows were never exposed to insecticidal drugs, nor did they receive antibiotics or growth hormones. The composition of the powder of colostrum was as follows (w/w): 84.7% protein, <8.4% fat, 7.0 mg/g lactoferrin, and 327.7 mg/g immunoglobulin. Antibodies, detected by an indirect enzyme‐linked immunosorbent assay (ELISA) using plates coating carious microorganisms, were defined as positive when absorbance in ELISA for a 0.1% (v/w) powder solution was >1.0 and 4 times higher than that of skim milk. The IMMULAC™ powder contained antibodies against *Bacillus cereus*, *Campylobacter jejuni*, and *E. coli* including O157:H7 strain, *Helicobacter pylori*, *Klebsiella pneumoniae*, *Listeria monocytogenes*, *Propionibacterium acnes*, *Salmonella* Enteritidis, *Salmonella* Typhimurium, *Staphylococcus epidermidis*, *Streptococcus agalactiae*, *Streptococcus mutans*, *S. pneumoniae*, and *Yersinia enterocolitica* (Funatogawa et al., 2002). Skim‐milk powder was obtained from Meiji Seika Co. The powder was dissolved into sterile water plates (100 μg/ml). at a 5% solution. Streptomycin sulfate (Meiji Seika) was dissolved in drinking water (5 g/L) or nutrient agar plates (100 μg/mL).

### 2.3 Bacterial strains

Streptomycin‐resistant *E.* *coli* O157:H7 strain (O157‐SM^R^) (Fukuda et al., 1998) was provided by Dr. Yoshichika Arakawa, Nagoya University. These organisms were grown in brain heart infusion broth at 37 ℃ for 24 hr. The viable numbers of these organisms were determined using quantitative cultivation on nutrient agar plates supplemented with streptomycin (50 μg/ml) for O157‐SM^R^. *Salmonella enterica* serovar Enteritidis No. 11 F1 was provided by Dr. Masayasu Nakano, Jichi Medical School. These organisms were grown in brain heart infusion broth at 37℃ for 24 hr. *Mycobacterium avium* MINO was provided by Dr. Toshitaka Goto, Miyazaki University.

### 2.4 Infection protocols

Groups of mice (*n* = 5 or 6 per group) were given drinking water that did or did not contain streptomycin for 7 days before administrations of *E. coli* O157‐SM^R^ and *S. enterica* serovar Enteritidis No. 11 F1. The grown *E. coli* and *S.* Enteritidis organisms were washed by saline and suspended in saline. The bacterial suspensions (*E. coli*: 2 × 10^1^ to 2x10^6^, *S.* Enteritidis: 2.5 × 10^1^ to 2.5 × 10^6^ CFU/mouse) were administrated orally into mice. The IgG‐enriched whey fraction or skim milk was given ad libitum at a 5% solution instead of water from 1 hr after bacterial challenges. Mice infection with *E. coli* O157‐SM^R^ was performed as described previously (Funatogawa et al., 2002). The humane endpoint was set up as 20% body weight loss or inability to access food or water after bacterial infection.

Bacterial suspensions of *M. avium* MINO were administrated into mice at 40 μl of 1.6 × 10^4^ CFU/mouse intranasally after anesthetizing with 400 μl of 2.5% tribromoethanol intraperitoneally. After administration, drinking water was replaced by a solution containing the IgG‐enriched whey fraction or skim milk. Lungs were removed from mice at 1, 2, and 3 weeks after infection, and homogenized, and then viable numbers of *M. avium* in the homogenate were determined using Middlebrook 7H11 agar (Difco Laboratories, MS).

### 2.5 Statistical analysis

Statistical significance was determined by Student's *t* test for bacterial counts and by the chi‐square test for mortality.

### 2.6 Ethics approval

Ethical approval was obtained from Ethical Committee of Tochigi Prefectural Institute of Public Health and Environmental Science (approval numbers: H30‐000731).

## 3 RESULTS

### 3.1 Chemical and bacterial analysis of IgG‐enriched milk

The IgG‐enriched whey fraction contained 84.7% _m/m_ of protein, 8.4% _m/m_ of fat, 0.9% _m/m_ of lactose, and 5.2% _m/m_ of moisture. It contained 327.7 mg/g of IgG, 7.0 mg/g of lactoferrin, and 10.0 mg/g of sIgA. In the enriched‐IgG whey fraction, *Bacillus cereus*, *Clostridium perforingens*, Coagrase‐positive *staphylococcus* species, *Enterobacteriaceae*, *Coliform* bacteria, *E. coli*, *E. coli* O157:H7, *Listeria* species, *Cronobater* species, and *Salmonella*  species were not detected.

### 3.2 Effects of pretreatment of streptomycin on *Salmonella* Enteritidis infection

Mice were given drinking water with streptomycin (5 μg/ml) or without streptomycin for 7 days. After given nothing per os for 7 hr, *S.* Enteritidis was orally inoculated in mice at indicated doses. After inoculation, these mice were given drinking water without streptomycin and fed normally, and were observed for survival for 4 weeks (Table 1). The medium lethal doses (LD50 values) were 1 × 10^3^ CFU/mouse in streptomycin‐pretreated mice and >1 × 10^6^ CFU/mouse in nontreated mice (Table 1).

**Table 1:** Effects of streptomycin (SM) pretreatment on susceptibility to *S.* Enteritidis in mice

	Survival (alive/total)^a^	
Inoculating doses (CFU)	SM‐pretreated mice (alive/total)	No treated mice (alive/total)
1.0 x 10^3^	5/5	5/5
1.0 x 10^4^	0/5	3/5
1.0 x 10^5^	0/5	4/5
1.0 x 10^6^	0/5	3/5

a Mice were given drinking water with streptomycin (SM; 5 μg/ml) or without SM for 7 days. *S.* Enteritidis was orally inoculated in mice at indicated doses. After inoculation, these mice were given drinking water without streptomycin and fed normally, and were observed for survival for 4 weeks.

### 3.3 IgG‐enriched fraction protect mice to *S.* Enteritidis and EHEC infection

**Figure 1:**
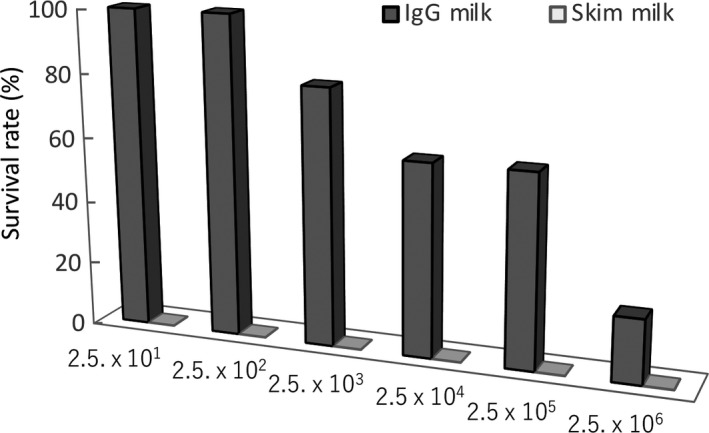
Protective effects of the IgG‐enriched whey fraction (IgG25^+^) against *S.* Enteritidis infection in mice. Mice were given drinking water that contained streptomycin for 7 days before administrations of S. Enteritidis No. 11 F1. *S.* Enteritidis was administrated orally into mice (2.5 × 10^1^ to 2.5 × 10^6^ CFU/mouse). The IgG‐enriched whey fraction (in dark gray) or skim milk (in light gray) was given ad libitum at a 5% solution instead of water from 1 hr after bacterial challenges. The humane endpoint was set up as 20% body weight loss or inability to access food or water after bacterial infection

When inoculated 2.5 × 10^1^ or 2.5 × 10^2^ CFU of *S.* Enteritidis in mice, all mice survived in the group of the IgG‐enriched whey fraction administration (Figure 1). The survival rates of mice decreased in the group in the dose‐dependent manner of the numbers of inoculated *S.* Enteritidis from 2.5 × 10^3^ to 2.5 × 10^6^ CFU/mouse, with LD_50_ of 5 × 10^5^ CFU/mouse (Figure 1), whereas no mouse survived in the group of skim‐milk administration when inoculated from 2.5 × 10^1^ to 2.5 × 10^6^ CUF/mouse, with LD_50_ of< 2.5 × 10^1^ CUF/mouse (Figure 1).

**Figure 2:**
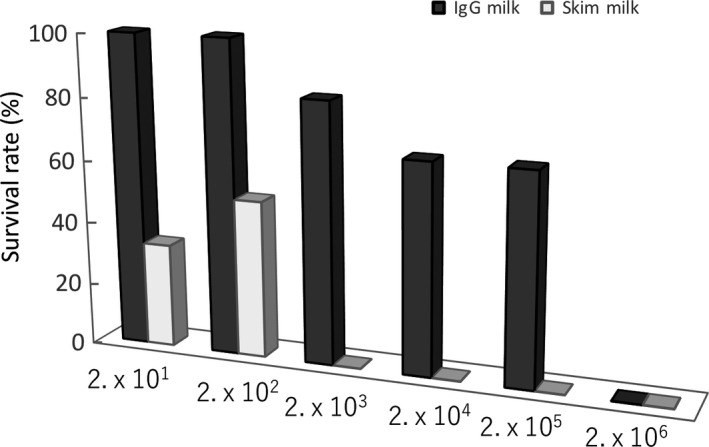
Protective effects of the IgG‐enriched whey fraction (IgG25^+^) against *Escherichia coli* O157‐SM^R^ infection in mice. Mice were given drinking water that contained streptomycin for 7 days before administrations of *E. coli* O157‐SM^R^. *E. coli* was administrated orally into mice (2 × 10^1^ to 2 × 10^6^ CFU/mouse). The IgG‐enriched whey fraction (in dark gray) or skim milk (in light gray) was given ad libitum at a 5% solution instead of water from 1 hr after bacterial challenges. The humane endpoint was set up as 20% body weight loss or inability to access food or water after bacterial infection

When inoculated 2 × 10^1^ or 2 × 10^2^ CFU of EHEC in mice, all mice survived in the group of the IgG‐enriched whey fraction administration (Figure 1). The survival rates of mice decreased in the group in the dose‐dependent manner of the numbers of inoculated *S.* Enteritidis from 2 × 10^3^ to 2 × 10^6^ CFU/mouse, with LD_50_ of 4 × 10^5^ CFU/mouse (Figure 2). When inoculated 2 × 10^1^ and 2 × 10^2^ CFU of EHEC in mice, 2 of 6 and 3 of 6 mice survived in the group of skim‐milk administration, respectively (Figure 1). No mouse survived in the group of skim‐milk administration when inoculated from 2 × 10^3^ to 2 × 10^7^ CUF/mouse, with LD_50_ of<1.5 × 10^2^ CUF/mouse (Figure 2).

### 3.4 IgG‐enriched fraction inhibited the growth of *M. avium* in lungs of mice

**Figure 3:**
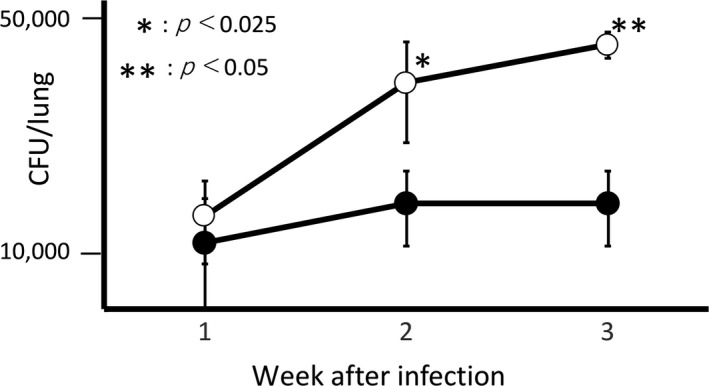
* Mycobacterium avium* MINO was administrated into mice 1.6 × 10^4^ CFU/mouse intranasally. After administration, drinking water was replaced by a solution containing the IgG‐enriched whey fraction or skim milk. Lungs were removed from mice at 1, 2, and 3 week(s) after infection, and viable numbers of *M. avium* in lungs were determined

When inoculated with 1.6x10^4^ CFU/mouse of *M. avium*, the numbers of CUF in lungs did not increase from 1 to 3 weeks after inoculation in the group of the IgG‐enriched whey fraction administration, whereas the numbers of CFU in lungs increased from 1 to 3 weeks after inoculation in a time‐dependent manner in the group of skim‐milk administration (Figure 3). As the results, the numbers of CFU in lungs in the group of the IgG‐enriched whey fraction administration were significantly smaller than those in the group of skim‐milk administration.

The IgG‐enriched preparations did not show direct bactericidal effects in vitro, when *S.* Enteritidis and EHEC were incubated with more than 7% of the IgG‐enriched whey fraction solutions (data not shown).

## 4 DISCUSSION

Bovine IgG‐enriched preparations protect bacterial infections on several levels via enhancing clearance of pathogens, preventing colonization, and modulating immune functions (Funatogawa et al., 2002; Ulfman et al., 2018). Our previous in vitro study using sections made from the cecum wall demonstrated that 5% solution of immunoglobulin‐enriched bovine colostrum significantly blocked the attachment of EHEC O157 to mucous membrane and inhibits the colonization of the bacteria (Funatogawa et al., 2002).

This is the first report that oral administration of bovine IgG‐enriched preparations suppressed the respiratory tract infection caused by *M. avium* in mice, although several animal studies have shown effects of bovine IgG and colostrum in viral respiratory tract infections, including respiratory syncytial virus infection in mice (Xu, Kim, Wi, & Kim, 2015) and influenza virus infection in mice (Ng, Wong, Muller, Rawlin, & Brown, ). An early study demonstrated that 0.7% of 125I‐labeled bovine IgG was uptaken by intestinal tracts of adult rats, when intestinally administrated. The data suggest that bovine IgG uptaken via intestinal tracts directly inhibit the growth of *M. avium*. Interferon‐γ production plays a major role of immunity against infection with *M. avium* as well as *Mycobacterium tuberculosis* (van de Vosse, Hoeve, & Ottenhoff, 2004). The protective effects, therefore, would be explained by the activation of intestinal immunity against *M. avium* by bovine IgG.

## 5 CONCLUSION

Oral administration of a bovine IgG‐enriched whey fraction protects mice against food‐borne infections with Enterohaemorrhagic *Escherichia coli* O157:H7, *Salmonella enterica* serovar Enteritidis, and *Mycobacterium avium*, and it partially protects mice against respiratory tract infection.

## CONFLICT OF INTEREST

M.T. works for Aotearoa Co., Ltd.

## ETHICAL STATEMENT

The protocols and procedures of animal experiments in this study were ethically reviewed and approved by Ethical Committee of Tochigi Prefectural Institute of Public Health and Environmental Science (approval numbers: H30‐000731). This work does not involve any human studies.
